# Rice Bran Polysaccharides: Structure, Modification, Bioactivity, and Application Potential in Food and Health Systems

**DOI:** 10.3390/foods15122194

**Published:** 2026-06-17

**Authors:** Jiayue Li, Yuanyuan Sun, Mengran Zhang, Mengjia Chen, Hongzhi Liu, Xuchun Zhu, Feiyue Ren, Linyi Zhou, Zhongjiang Wang

**Affiliations:** 1 Key Laboratory of Geriatric Nutrition and Health, College of Food and Health, Ministry of Education, Beijing Technology and Business University, Liangxiang, Fangshan District, Beijing 100048, China; 15103323619@163.com (J.L.); sunyuanyuan0131@126.com (Y.S.); 19855856955@163.com (M.Z.); 13148008906@139.com (M.C.); liuhz0700@btbu.edu.cn (H.L.); xxcy08@163.com (X.Z.); 20200101@btbu.edu.cn (F.R.); 2 College of Food Science, Northeast Agricultural University, Xiangfang District, Harbin 150006, China; wzjname@126.com

**Keywords:** rice bran polysaccharide, nutritional function, functional properties, industrial production

## Abstract

Rice bran polysaccharides (RBPs) are natural plant-derived polysaccharides predominantly found in the rice bran layer, a major byproduct of rice milling, and exhibit a wide range of biological activities. These bioactive components, with their complex structures and diverse functions, hold immense potential for application in the food, pharmaceutical, and cosmetic industries. This review summarizes recent advances in the composition and bioactive functions of RBPs, with particular emphasis on how extraction methods and physicochemical modifications alter their molecular weight, monosaccharide composition, and chain conformation, thereby modulating key biological activities, including anti-inflammatory, antioxidant, immunomodulatory, and gut microbiota-regulating effects. Furthermore, to better elucidate their potential in industrial applications, this study systematically analyzes how the physicochemical properties of rice bran polysaccharides influence production and processing, with particular attention to their emerging roles as delivery carriers, food additives, and bioactive components.

## 1. Introduction

According to data from the Food and Agriculture Organization (FAO), China’s rice production in 2024 was 207 million tons, accounting for 20.10% of the world’s total rice production [1]. Rice is a staple food worldwide and contributes approximately 20% of the daily caloric intake of the global population [2]. Sabyasachi [3] points out that rice is the only commodity crop capable of feeding half the world’s population. However, the rice milling process generates large amounts of rice bran, most of which has not been fully utilized. Traditionally, rice bran has been used mainly as animal feed, incinerated, or directly discarded, leading to substantial resource loss. Rice bran is rich in various beneficial bioactive compounds. Increasing attention has therefore been directed toward the valorization of rice bran as a source of health-promoting bioactive compounds. Consequently, an increasing number of industries are turning their attention to rice bran [4].

RBPs are the primary components of rice bran. RBPs offer numerous health benefits. They help regulate blood sugar and blood lipids, possess antibacterial and antioxidant properties, aid in tumor suppression, and strengthen the immune system. Recent studies indicate that arabinogalactan in rice bran exhibits anti-inflammatory effects in laboratory tests [5]. The higher the arabinose content in arabinogalactan, the stronger its immunomodulatory effects [6]. Modifying RBP with surfactants can enhance its hydrophilic and lipophilic properties [7]. The triple-helix structure helps RBP activate immune receptors [8]. RBP can also serve as a substitute for synthetically produced nutrients, contributing to the maintenance of human health [9]. In recent years, RBP has become a hot topic in food and pharmaceutical research due to its wide-ranging applications in health and immune health [10]. At the same time, efforts are underway to produce RBP products on an industrial scale, such as RBP tablets and RBP-iron liposomes [11,12]. However, our understanding of the mechanisms of action of RBP at the microscopic level remains incomplete, and the methods for achieving large-scale production are not yet fully understood [13,14]. Therefore, a comprehensive analysis of the physicochemical properties and biological activities inherent to RBP is of paramount importance for the development of RBP-based food additives and pharmaceuticals.

## 2. Classification and Composition of RBP

**Figure 1:**
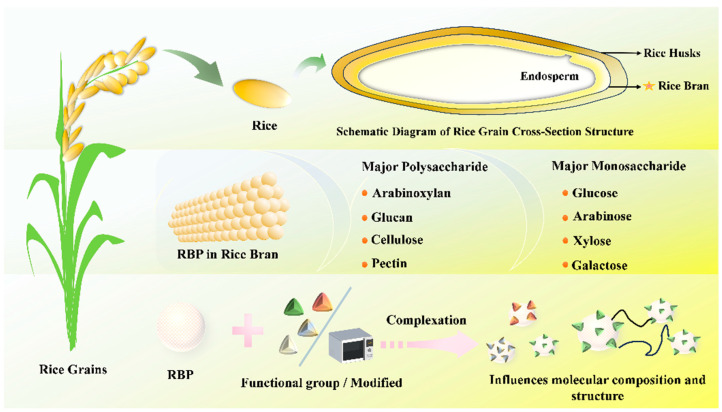
Schematic diagram of molecular composition and structural changes in RBP.

RBP is not a single molecular component, but rather a complex mixture of non-starch polysaccharides derived from the bran layer of rice grains [15]. Its major polysaccharide components include xylan [16], glucan [17], cellulose [18], pectin [19], and trace amounts of lipopolysaccharides [20] and glycoproteins [21] (Figure 1). Among these, the xylans in rice bran exist almost entirely in the form of arabinoxylans. In contrast to the native RBP mixture, RBAC (rice bran arabinoxylan compound) represents a functional complex enriched in arabinoxylan [22], and MGN-3 (Biobran) is an enzymatically modified arabinoxylan derived from RBP [23]. Together with glucans, they are the most extensively studied soluble components due to their diverse biological activities, while cellulose and pectin are primarily retained in the extraction residue as insoluble dietary fiber [24]. According to their chemical composition, RBPs are categorized as non-starch polysaccharides, which are heteropolysaccharides connected by glycosidic bonds, distinct from starch [25]. With regard to their physical properties, RBPs cannot be straightforwardly classified as either soluble or insoluble polysaccharides; their classification is primarily contingent upon solubility characteristics [26]. The extraction method employed significantly influences the composition of the RBP fraction, typically resulting in a heteropolysaccharide rich in arabinan and containing varying proportions of glucan and pectin polysaccharides [17]. Extracts derived through aqueous extraction are primarily composed of soluble non-starch polysaccharides, whereas those obtained via alkaline extraction are mainly composed of insoluble non-starch polysaccharides [17]. Chen et al. [27] utilized the alcohol precipitation method to further separate and purify RBPs into soluble non-starch polysaccharides. As a result, differences in extraction methods not only determine the solubility classification of RBP but also have a profound impact on its monosaccharide composition and molecular structure. Meng [28] demonstrated that, compared to traditional hydrothermal extraction of RBP, alkaline extraction combined with ultrasonic–microwave synergistic treatment can significantly increase xylose yield, thereby influencing the final polysaccharide content of RBP. Furthermore, fermentation treatment can alter the polysaccharide composition of RBP. As fermentation time increases, the content of xylose and arabinose gradually decreases and eventually disappears [29].

Determining the monosaccharide composition is fundamental to the structural characterization of RBP. In fact, whether it involves optimizing extraction processes, structural modifications, or characterizing biological activity, it is essential to determine the monosaccharide composition of RBP and, in some cases, the relative molar ratio of uronic acids [30] (Table 1). This is because the monosaccharide composition directly reflects the presence and relative proportions of different polysaccharide components. Chen’s analysis [31] of the monosaccharide composition indicated that RBP revealed the absence of reducing sugars, glucuronic acid, and starch, while identifying the presence of glucose, arabinose, xylose, and galactose [21,32]. Furthermore, changes in the monosaccharide profile under different extraction or modification conditions (such as fermentation, sonication, or chemical derivatization) are often used as indicators of structural alterations [28,29,33]. Studies on the glycosidic bond structure of RBP have identified two predominant types: α-1,4 bonds, which form the linear backbone, and α-1,6 bonds, which act as branching points, resulting in a highly branched architecture [21]. These glycosidic bonds collectively contribute to the water solubility of RBP. The biological activity of RBP is intricately linked to the specific types of monosaccharides, their relative proportions, and the molecular weight of the RBP [34]. Direct evidence supports the impact of monosaccharide composition on the functionality of RBP. Zha et al. [35] revealed that the PW2 fraction, isolated from RBP and devoid of mannose, generally exhibits reduced antioxidant activity.

Furthermore, research indicates that RBP predominantly adopts an α-conformation, although it also contains a minor proportion of β-conformations, which are implicated in immune regulation [38]. Among polysaccharides derived from various sources, those with high molecular weight facilitate the formation of effective cross-linked networks with immune receptors [39], whereas low-molecular-weight polysaccharides are more readily metabolized by the gut microbiota to perform prebiotic functions. For RBP, strategic modification not only enhances its molecular weight but also augments its functional properties [40]. Techniques such as fermentation and co-extrusion can decrease the molecular weight of RBP, potentially altering its antioxidant properties [41]. Conversely, chemical modification and cross-linking can increase the molecular weight of RBP through covalent bonding, thereby broadening its industrial applications [42]. Therefore, although monosaccharide analysis is widely adopted as an essential primary characterization method, it is typically necessary to combine it with studies of glycosidic linkage types, molecular weight distribution, and chain conformation to fully understand the structure–activity relationship of RBP.

### 2.1. Effect of Physical Modification on the Structure of RBP

**Figure 2:**
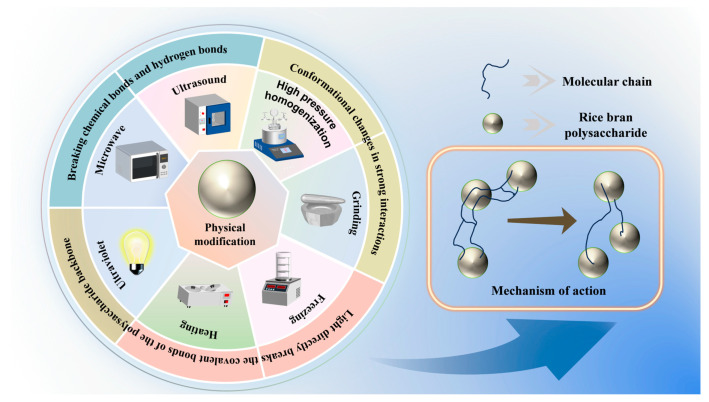
Effect of physical modification on RBP.

Studies have shown that physical modification can improve its solubility, stability, and bioactivity (Figure 2). The use of microwaves and irradiation can break down the cell walls of rice bran to elute more non-starch polysaccharides, and to some extent sever the glycosidic bonds tightly binding cellulose and hemicellulose, thereby increasing the elution rate of RBP and resulting in a greater variety of polysaccharide structures [43]. Infrared irradiation not only disrupts the cell walls of rice bran but also degrades the glycosidic bonds in the RBP chains to some extent, thereby altering the composition and content of monosaccharides. Yan et al. [33] discovered that infrared irradiation treatment increases the content of rhamnose, mannose, and glucose. Meanwhile, techniques such as sonication and microjet treatment can influence the local spatial conformation of polysaccharides. Ultrasonic treatment of RBP relies on cavitation-induced high shear forces and thermal effects to fracture, reorganize, and oxidize molecular bonds, altering monosaccharide composition and structure [36]. In addition, ultrasonic treatment can disrupt the crystalline regions of rice bran fibers, promoting disorder and reducing the molecular weight of polysaccharides [44]. Likewise, Suriano [45] found that microjet technology can alter the particle size distribution of cereal polysaccharides, significantly reducing their molecular weight. Furthermore, this technology disrupts crystalline regions within the polysaccharides, lowering their crystallinity and resulting in material with a looser structure. Subsequently, Zhong [46] successfully applied microjet technology to RBP, achieving efficient preparation of nanoscale RBP materials.

### 2.2. The Effect of Chemical Modification on the Structure of RBP

Multiple chemical modification methods are available for RBP, such as sulfation, carboxymethylation, acetylation, phosphorylation, and metal complexation (Table 2). These methods alter the structure of RBP and confer specific physicochemical properties, thereby improving or modulating its functionality.

#### 2.2.1. Effect of Sulfation Modification on the Structure of RBP

Sulfation modification involves the reaction primarily introducing sulfate groups at the primary hydroxyl group at the RBP of C-6 position [51]. This modification is closely related to the degree of substitution [52]. The solubility of the modified RBP in solution was improved, and its molecular weight increased significantly. Concurrently, the introduction of sulfate groups increases the negative charge on the RBP surface, which not only enhances its solubility in water but also enables targeted binding to positively charged bioactive substances [53]. Furthermore, following sulfation modification, the structure of RBP transformed from a rough surface to a smooth, coiled conformation, which facilitates the exposure of more active sites, thereby enhancing the recognition capacity of immune receptors [48].

#### 2.2.2. Effect of Carboxymethylation Modification on the Structure of RBP

Carboxymethylation modification involves the introduction of carboxymethyl groups into the molecular chain of the RBP molecule. This process first induces significant changes at the structural level. A study on millet bran polysaccharides by Zheng et al. [54] showed that carboxymethylation disrupts dense internal structures, forming a porous microstructure, which may inform similar modifications in RBP. Huang et al. [55] identified two characteristic infrared peaks (1603 cm^−1^ and 1329 cm^−1^) in carboxymethylated RBP, corresponding to the asymmetric stretching vibration of the ionized carboxylate group (-COO^−^). The presence of these peaks confirms the successful grafting of carboxymethyl groups onto the RBP backbone. These structural alterations further induce physicochemical changes, including increased molecular negative charge, enhanced hydrophilicity, and reduced crystallinity due to hydrogen bond network reorganization [42].

#### 2.2.3. Effect of Metal Chelation on the Structure of RBP

The chelation of polysaccharides with metals induces specific structural changes in RBP chains, enhancing the stability and biological activity of the chelating complexes. For RBP, numerous active groups are distributed along the molecular chains, providing a large number of binding sites for metal ions. Liu initiated Fe ion chelation with RBP, successfully producing stable Fe-RBP complexes [50]. Subsequently, Chen applied this Fe-RBP to liposome encapsulation [11]. To investigate structural changes in this stable chelate, Pan [56] systematically analyzed RBP chelation with various metal ions including Ca, Cu, Zn, and Fe for the first time. They discovered that diverse metal ions form coordinate bonds with oxygen atoms in the hydroxyl or carboxyl groups of RBP, successfully chelating them to the polysaccharide. For RBP, different metal ions impart distinct functional effects. Among the four components studied, only iron-bound RBP exhibited potent antioxidant properties, effectively inhibiting ferrous ions from participating in oxidation. Therefore, further exploration of novel conformations for metal ion chelation-modified RBP remains essential to advance its applications in food, pharmaceutical, and other fields.

#### 2.2.4. Effect of Other Modifications on the Structure of RBP

Beyond established RBP modification methods such as sulfation and carboxymethylation, several RBP modifications remain under exploration. Park [52] demonstrated that the free primary hydroxyl group at position C6 in RBP exhibits chemical selectivity, enabling oxidative modification (-OH oxidation to -COOH) and sulfation (-OH sulfation to -OSO_3_^−^) at this site. Lovegrove [57] further identified the C5 hydroxyl group of arabinose residues as a specific site for feruloylation. Huang et al. [55] substituted hydroxyl groups with phosphate groups to obtain phosphate ester derivatives, exhibiting characteristic P=O peaks at 1264 cm^−1^ in infrared spectroscopy and increased molecular negative charge density. Li [58] observed that acetylation modification alters polysaccharide chain conformation, exposing more polar groups and improving the amorphous regions of the polysaccharide structure. However, the applicability of this modification method is constrained by the chemical structure of RBP, resulting in low acetylation substitution levels, increased costs, and significant differences compared to carboxymethylated RBP [55]. In summary, although phosphorylation and ferulylation are still in the exploratory phase, they have established specific sites and characterization methods, laying the foundation for precisely constructing novel RBP functional materials.

## 3. Functional Properties of RBP

Research indicates that RBP exhibits a range of functional characteristics, including antioxidant, antibacterial, anti-inflammatory, antitumor, anticancer, anti-radiation, hypoglycemic, blood-lipid-lowering, and immune-boosting activities, as well as serving as a delivery carrier [25,59]. Additionally, RBP demonstrates diverse processing functionalities, such as enhancing emulsification [60], stability, water retention, rheological properties [61], and gene transfection performance [62]. These findings are of considerable importance for the exploration of RBP, providing a foundation for the development of functional foods, pharmaceuticals, cosmetics, and health supplements derived from RBP.

### 3.1. Biological Functions of RBP

The nutritional functions of RBPs are intrinsically linked to their structural characteristics, including molecular composition, glycosidic bond configuration, and molecular weight [63]. The type and position of glycosidic bonds significantly influence the anti-inflammatory activity of polysaccharides. Specifically, the β-(1→6) glycosidic bonds in glucan exhibit immune activity by recognizing the Dectin-1 receptor [64]. Sulfation studies of purple RBP have demonstrated that this process induces desulfation phenomena with quantifiable degrees of substitution (DS), which subsequently activate macrophage responses, thereby facilitating immunomodulation in vivo [13]. Furthermore, the immune activity of RBP can be enhanced by modifying the substituent groups through chemical methods such as acetylation, selenylation and carboxymethylation [55].

#### 3.1.1. Anti-Inflammatory

**Figure 3:**
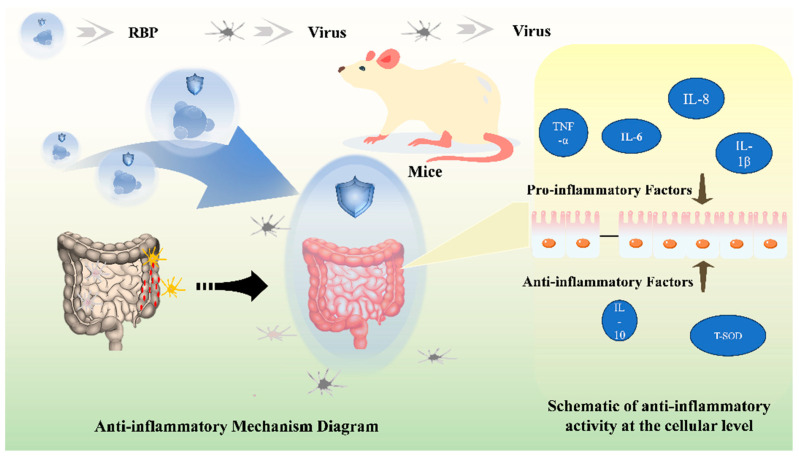
Schematic diagram of the anti-inflammatory function of RBP.

The anti-inflammatory mechanism of RBP primarily involves two pathways: promoting the expression of anti-inflammatory factors and suppressing the expression of pro-inflammatory factors (Figure 3). Surin [13] demonstrated that RBP exerts anti-inflammatory effects by suppressing mRNA expression of the pro-inflammatory factor iNOS. Additionally, Li [65] observed that after RBP administration, diseased mice exhibited significantly elevated levels of anti-inflammatory factors (IL-10 and T-SOD), promoting anti-inflammatory factor expression and alleviating DSS-induced chronic colitis. Fadel [5] found that enzymatically hydrolyzed arabic xylan in RBP significantly activated NK (natural killer) cells and macrophages, exhibiting pro-inflammatory and immune-enhancing effects. Conversely, non-enzymatically treated samples selectively inhibited IL-4, demonstrating classical anti-inflammatory activity. The aforementioned studies indicate that different components within RBP can regulate immunity through unique pathways.

#### 3.1.2. Antioxidant

The antioxidant capacity of RBP is influenced by factors such as rice variety and extraction method. Surin [66] compared seven Thai rice bran varieties and found that the Kum Doi Saket variety exhibited the highest polysaccharide content and antioxidant activity (*p* < 0.05). They also incidentally discovered significant differences in RBP antioxidant activity when using different organic solvents for extraction, with hexane-extracted RBP demonstrating superior antioxidant properties. This may be attributed to the defatting step enhancing the purity of antioxidant components. In terms of the mechanism of action, Ghoneum [67] and Surin [68] demonstrated that RBP reduces pro-inflammatory factors (IL-6), decreases intercellular adhesion molecule-1 (ICAM-1), and activates the Nrf2/ARE antioxidant pathway. Moreover, RBP can modulate the activity of luciferase through antioxidant reactions and the activity of antioxidant factors NQ01 and HO-1 [69]. Subsequently, Liu [70] purified RBP using a gradient ethanol precipitation method. The results demonstrated a linear positive correlation between antioxidant activity (DPPH, FRAP) and purity, providing the first data-driven graphical representation of this relationship. To validate RBP’s in vivo protective effects, Elbaghdady et al. [67,71] demonstrated that the MGN-3 fragment in RBP significantly reduces protein plaques in mouse brains, confirming that RBP’s antioxidant effects also exert neuroprotective functions. However, other polysaccharides within RBP require further isolation, purification, and validation to establish the relationship between the various components of RBP and antioxidant activity.

#### 3.1.3. Anticancer, Antitumor

In terms of anticancer mechanisms, studies have shown that various bioactive polysaccharides derived from rice bran also possess significant antitumor and immunomodulatory functions [72]. In mouse models, supplementation with MGN-3 can enhance tumor cell death through the apoptosis mechanism [73]. Furthermore, it enhances the activity of NK cells, thereby aiding in the recognition and killing of cancer cells [73]. Another study indicated that enzymatically modified RBP significantly enhances the cytotoxicity of NK cells against K562 leukemia cells. However, the magnitude of this increase is relatively small, at approximately 12% [74]. The physiological significance of this effect remains to be further determined. However, direct evidence regarding its role in cancer prevention in humans remains limited, and further clinical studies are required. With regard to anticancer activity in vitro, although the polysaccharide extracted from bioprocessed black rice bran does not directly reduce cancer cell viability in triple-negative breast cancer cells or in those resistant to radiotherapy, it significantly inhibits clonogenic formation and reduces cancer cell adhesion to endothelial cells, as well as endothelial cell migration and invasion [75]. Therefore, BRP may exert its anticancer effects indirectly by modulating the tumor microenvironment, rather than through direct cytotoxic action. However, all results in this study are derived solely from cell experiments; further in vivo animal studies and human trials are needed to confirm these effects and evaluate their potential for practical application.

RBP exerts antitumor effects through multiple mechanisms, including reducing adhesion between cancer cells and endothelial cells, promoting cancer cell apoptosis, enhancing immune function, and blocking tumor progression by interfering with virus–host receptor interactions [10]. Mukherjee [76] found that negatively charged sulfated polysaccharides can competitively bind to viral proteins, thereby blocking their interaction with host heparan sulfate receptors. This advancement provides new insights into the antitumor mechanisms of RBP modified by sulfation, carboxymethylation, and other processes. Furthermore, fermented RBP exhibits significant advantages in antitumor activity. RBP extracted from Ganoderma lucidum fermentation resulted in an 87.59% relative reduction in tumor volume compared to the control group [29].

#### 3.1.4. Anti-Radiation

In terms of radiation protection, several preclinical studies have investigated polysaccharides extracted from rice bran, particularly MGN-3 effectively preventing radiation-induced intestinal barrier dysfunction by maintaining the activity of mitochondrial respiratory chain complexes in the duodenal and colonic mucosa [23]. Ghoneum [77] observed that in a mouse model of whole-body γ-radiation exposure, pretreatment with MGN-3 significantly reduced radiation-induced weight loss and alleviated anemia and leukopenia. Furthermore, bone marrow cell activity and spleen size were preserved. However, the current evidence for radioprotection is limited to murine models and the modified derivative MGN-3 and direct evidence for native RBP in humans is lacking.

#### 3.1.5. Antibacterial

The diameter of the inhibition zone around pathogenic bacteria serves as an indicator for assessing antimicrobial performance. Regarding the antibacterial activity of RBP, Surin [58] demonstrated that unmodified RBP effectively inhibits pathogens such as Staphylococcus aureus and *Escherichia coli*. Meanwhile, Chen [78] found that modified RBP can also significantly disrupt the cell walls of Gram-positive bacteria. Furthermore, metal ion chelation modification can enhance the antibacterial efficacy of RBP [79]. Neha Khan [80] successfully chelated polysaccharides with iron complexes, achieving exceptional stability. This chelate significantly enhanced antioxidant and anti-hemolytic activities. However, further experimental validation is required to confirm whether chelates prepared via chelation technology possess superior antibacterial properties and in vivo green safety. In summary, while RBP demonstrates significant in vitro antibacterial effects, in vivo safety must be prioritized to advance the development of RBP-based food additives, pharmaceuticals, and health supplements.

#### 3.1.6. Lowering Blood Sugar and Blood Lipids

Blood sugar is primarily formed through gluconeogenesis, metabolic pathways in the body, and external intake through digestion and absorption. Blood lipids are primarily produced through intake by the liver. RBPs are water-soluble polysaccharides that can inhibit the activity of α-glucosidase and α-amylase in the intestines, thereby lowering blood sugar and blood lipids [21]. Rahim et al. [81] concluded that fructooligosaccharides in rice bran can improve the intestinal flora, enhance mineral absorption in the intestine, and lower cholesterol, triglyceride, and phospholipid levels, thereby achieving hypoglycemic and hypolipidemic effects. Tan et al. [82] found that intake of a lipopolysaccharide dietary supplement pretreated with rice bran significantly reduced plasma total cholesterol and triglyceride levels of mice lacking low-density lipoprotein receptors on a high-fat diet. In addition, RBAC positively regulates serum glucose, lipid, and protein metabolism in diabetic patients [22].

#### 3.1.7. Immunomodulatory Activity

**Figure 4:**
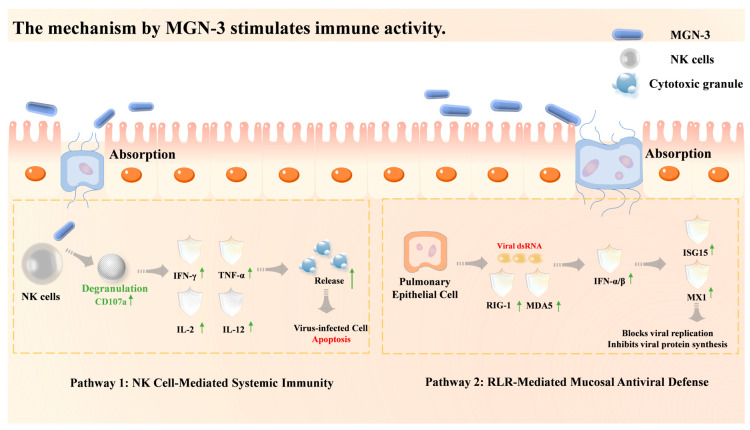
The mechanism by MGN-3 stimulates immune activity.

Currently, arabinoxylan in RBP is a widely studied functional plant polysaccharide health supplement. As reported by Ahmed [83], dietary supplementation with MGN-3 derived from rice bran significantly enhances cellular natural killer activity against influenza viruses, thereby boosting immunity. Another mechanism involves enhancing the antioxidant capacity of immune cells, thereby influencing cellular immune responses and boosting the secretion of immune response substances to improve the efficiency of the immune system (Figure 4). Yuandani [84] used rice glycoproteins as an example, demonstrating enhanced immunomodulatory functions by inducing spleen lymphocyte proliferation and increasing cytokine and nitric oxide production. Furthermore, Liu [74] demonstrated that fermented RBP modified by Grifola frondosa enhanced immune responses in diseased mice by increasing NK cell activity and improving overall immune efficiency. Similarly, in the study by Kang, the immune response following treatment with RBP fermented by shiitake mushroom mycelium was observed [85]. RBP demonstrated the ability to stimulate NK cells in mice, elevate nitric oxide and cytokine levels, and enhance the activity of lysosomes in splenic cells and peritoneal macrophages. This approach strengthened the body’s defense against viral invasion and bolstered immune function.

#### 3.1.8. Delivery Carrier

RBP has been explored as a potential carrier for drug and bioactive compound encapsulation due to its biocompatibility and modifiable structure [86]. Currently, the main polysaccharide delivery carriers for rice bran include: hydrogels [87], nanoparticles, microcapsules, films, hydrogel beads, electrospun fibers, and liposomes (Table 3). Stable carrier structures formed through physical or chemical methods can be used to encapsulate substances that are sensitive to environments such as gastric acid, thereby achieving protection, controlled release, and targeted delivery. However, most studies remain at the preclinical stage, and challenges such as in vivo stability, batch-to-batch consistency, and large-scale production need to be addressed before clinical translation.

Existing studies have utilized RBP to encapsulate DNA, siRNA, and fish oil, confirming that this carrier exhibits excellent sustained-release properties and targeted delivery capabilities at the cellular level and in animal models [7]. Wu [92] employed grafting technology to combine polyethyleneimine (PEI) with rice bran polysaccharide (RBP), creating modified rice bran polysaccharide (PRBP) that forms a carrier protecting DNA. Liu [93] further validated its high efficiency and biocompatibility in gene delivery. Furthermore, RBP-based packaging materials show broad application prospects in the food industry, as they can enhance water dispersibility, mask off-flavors, improve stability, and reduce volatility [78]. In the pharmaceutical field, they enable controlled, precise drug release and serve as delivery carriers [94]. Overall, the functional properties of these packaging materials stem from the network structure of polysaccharide chains, their tunable physical properties, and excellent biocompatibility, giving them broad application potential in both pharmaceutical and food sectors.

### 3.2. Functions of RBP

Driven by the increasing demand for multifunctional, bio-based natural components within the food and pharmaceutical sectors, RBP is garnering significant attention. Its distinctive physicochemical properties and biological activities confer substantial advantages in processing applications (Table 4). Functional studies emphasize the importance of examining RBP’s water-holding capacity to enhance food texture and prolong shelf life. Stability research is essential to ensure consistent performance across various processing conditions, while investigations into its ability to modulate hardness and viscosity reveal extensive potential applications in food and health supplements. Furthermore, research into the emulsifying and solubility properties of RBP contributes to the development of more stable emulsion systems. Chemical modification and the introduction of various functional groups can alter RBP’s solubility, viscosity, and biological activity. Comprehensive exploration of RBP’s industrial functions seeks to provide theoretical support and technical guidance for its application in the food, cosmetics, and pharmaceutical industries, thereby promoting its efficient utilization and fostering sustainable development.

#### 3.2.1. Emulsifying Properties

As one of the most abundant functional components in rice bran, RBP possesses a structure rich in hydroxyl and aldehyde groups, making it suitable for modification. However, unmodified RBP lacks sufficient surface activity, making it difficult to form an adsorption layer with high mechanical strength at the oil–water interface [60]. Therefore, it is necessary to enhance the emulsifying properties of polysaccharides through the synergistic effects of physical modification and functional group grafting, thereby meeting the requirements of RBP for specific food processing applications, such as nano-delivery carriers.

Researchers primarily employ physical and chemical processing methods to enhance emulsification properties. Liu et al. [36] used ultrasound, enzymes, and subsequent ultrasound to expose more hydrophilic sites on RBP, thereby creating an optimal environment for the formation of a stable oil–water interface. Ciccoritti [99] utilized ultra-micronization to reduce particle size, improve polysaccharide solubility, promote migration to the interface, and enhance emulsification properties of RBP. Chemical modification primarily enhances amphiphilicity by introducing hydrophobic groups and altering molecular weight and structure. For example, Cui et al. [7] introduced Octenylsuccinic anhydride (OSA) groups, which significantly improved the emulsifying activity and stability of RBP.

#### 3.2.2. Thermal Stability

Recent studies on RBP’s thermal stability primarily target weakly acidic-to-neutral environments. In such conditions, RBP’s properties or interactions improve system stability during heating or storage. Han [43] found that microwave treatment alters RBP’s structure, boosting its heat resistance. Yilmaz-Turan et al. [100] showed that acetylation reduces intermolecular movement and degradation, enhancing arabic polysaccharides’ thermal stability. Experiments indicate that cheese sauce with 1% RBP remains as stable at 45 °C as refrigerated samples at 5–7 °C [61]. Lee [101] reported that freeze-drying preserves RBP’s structure, maintaining its nanoporous form and resistance to collapse during thermal processing.

#### 3.2.3. Water-Holding Capacity

RBP itself possesses strong hydrophilicity and exhibits certain water-holding capacity. Enhanced water-holding capacity improves texture. To increase RBP’s water-holding capacity, extraction and purification processes are required. These may involve physical and chemical treatments that disrupt crystal structures, exposing more hydrophilic groups and thereby enhancing RBP’s water retention ability [102]. For instance, Jia et al.’s [103] experiments demonstrated that fermentation treatment can further enhance RBP’s water-holding capacity. Zadeike et al. [37] found that the synergistic effect of ultrasonication and enzymatic hydrolysis significantly increased the degree of breakage in RBP’s soluble polysaccharide components, improving the purity and uniformity of RBP’s structure, thereby enhancing its water retention and swelling capabilities. Similarly, Chen’s [104] experiments also demonstrated that ultrasonic-assisted enzymatic treatment significantly improved the water-holding capacity and solubility of soluble dietary fiber from rice bran.

#### 3.2.4. Rheological Properties

By modifying RBP, their rheological properties can be improved. This not only effectively reduces reliance on single hydrophilic colloids such as guar gum and xanthan gum but also provides a greener, more superior solution for enhancing rheological properties. In cheese sauce systems, using 0.5–1.0% (*w*/*w*) modified RBP in conjunction with conventional thickening–emulsifying composite stabilizers can significantly reduce phase separation rates in high-fat, low-pH environments [61]. Additionally, Roye et al. [105] observed that the viscosity of RBP improved after extrusion combined with steam bursting. In starch-based foods, adding 0.5–1.0% RBP enhances the peak viscosity and gel strength of rice starch, improving stability at high temperatures [97].

#### 3.2.5. Gene Transfection Performance Properties

Research on the gene transfection performance properties of RBP primarily involves using medicinal and edible plants such as Grifola frondosa and Ganoderma lucidum to assist in the fermentation and extraction of RBP. The polysaccharide yield of the composite can reach 4.67% and 7.64%, significantly higher than that of unfermented polysaccharides [29]. Additionally, RBP can undergo chemical modifications such as phosphorylation, carboxymethylation, and acetylation to enrich different functional groups [55,58,106]. In studies on small intestine digestion and absorption, RBP can form stable complexes with Fe^3+^, Zn^2+^, and Ca^2+^, thereby enhancing mineral absorption. This chelation method endows the chelate with properties that enable gene transfection and targeted delivery [98].

## 4. Industrial Applications of RBP

**Figure 5:**
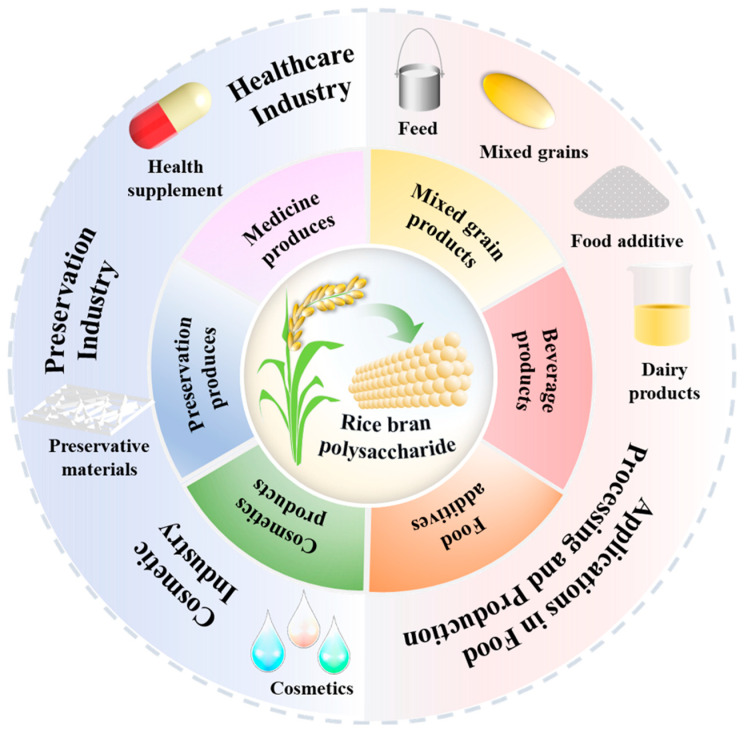
The application of RBP in industry.

In the food industry, RBP has been incorporated into baked products, dairy systems, food additives, and nutritional supplements to improve product quality and functional properties. Additionally, RBP is applied in industries beyond food production, such as packaging preservation, cosmetics, and biomedicine (Figure 5). The following sections will focus on analyzing the shortcomings and applications of RBP in industrial processes across different fields, aiming to enhance the application value of RBPs in various sectors and provide important reference for their future development.

### 4.1. Applications in Food Processing and Production

#### 4.1.1. Animal Feed Applications

In contemporary research, RBPs are increasingly acknowledged for their contributions to the quality of dietary baked goods. The existing body of literature investigates the application of RBP to improve both the texture and nutritional profile of these products. RBP can also be utilized as a feed additive. Luo [107] demonstrated that dietary supplementation with RBP significantly alleviated obesity induced by a high-fat diet. This supplementation was found to modulate gut microbiota and enhance the production of short-chain fatty acids in the colon, thereby reducing systemic inflammation and improving protein digestion and utilization in obese individuals. In the context of industrial production, the low-dose incorporation of non-starch polysaccharides can function as a prebiotic, enhancing digestive absorption in poultry [107]. Moreover, the transformation of insoluble polysaccharides into soluble forms, such as through carboxymethylation, markedly increases the nutritional value of RBP as a feed [108].

#### 4.1.2. Bakery and Cereal Products

In bread processing, RBPs are used as modifiers and colorants. The addition of RBP and extraction manipulation can improve dough performance. RBPs contain a large number of hydrophilic groups, such as hydroxyl groups, which can form hydrogen bonds with water molecules, thereby effectively absorbing and retaining moisture [109]. This property enables RBPs to significantly enhance water retention in foods, reduce moisture loss, and maintain the softness and juiciness of foods, particularly in baked goods and meat products [110]. Additionally, by improving water retention, RBP can reduce the free migration of moisture, thereby inhibiting the growth and reproduction of microorganisms and extending the shelf life of foods [96]. To improve product quality during the production of bread and other products, Huang et al. [111] found that adding low doses of insoluble arabinogalactan from RBP could enhance the gluten–starch network structure and increase the volume of baked bread. In addition, Park [52] improved the water solubility of RBP by chemically selectively oxidizing the primary hydroxyl group at the C6 position of the polysaccharides, and utilized this property in the industrial production of cookies and bread to enhance product quality. RBPs not only enhance nutritional value as functional substances but also improve the physicochemical properties of dough. However, in industrial applications, factors such as the complexity of processing methods, cost issues, and packaging and storage limit the large-scale production of RBP-based baked goods. Therefore, a balanced consideration of capital, efficiency, and nutrition is necessary to establish a new large-scale industrial chain development model.

#### 4.1.3. Functional Beverages and Yogurt Products

The combination of functional foods and beverages has been recognized, and RBPs, as prebiotics, have broad application potential and diverse sources. In the development of green health drinks, soluble dietary fiber, as a beneficial polysaccharide, has the ability to improve the functional properties of yogurt [112]. Regarding beverage applications, Kwon observed that RBP consumption in mice demonstrated significant protective effects against alcoholic liver injury [113]. Furthermore, Hansawasdi [114] conducted sensory evaluations of RBP-based cereal beverage formulations and found that such formulations did not adversely affect consumer acceptance. During beverage industry processing, researchers discovered that manufacturing foods under low-pH conditions minimizes RBP loss due to hydrolysis, thereby preserving its functional activity. However, in the industry-specific research and development of specialty beverages, most studies have focused on combining other rice processing byproducts with fermenting bacteria, while there are few reports on the combined application of RBP and fermenting bacteria. This situation has hindered the development of RBP beverages. Research indicates that the rational utilization of different bacterial strains in fermentation can alter the composition of polysaccharides in grains, which is beneficial for the development and utilization of specialty beverages [115].

#### 4.1.4. Food Additives

RBPs are utilized in food additives for preservation purposes or to enhance food texture. In industrial production, the incorporation of polysaccharides into flour functions as a natural thickening agent for food and beverages, thereby reducing the need for additional refined ingredients [116]. The application of RBP in texture modification is the most extensively explored area in its use as a food additive. As a stabilizer in complex matrices, Chu et al. demonstrated the application of modified RBP as a stabilizer in complex matrices, specifically in cheese sauce [61]. When combined with conventional emulsifiers, the modified RBP decreased phase separation by 40% during accelerated storage. This advancement has notably enhanced product quality and contributed to the stabilization of oil-rich, acidic condiments during storage and transportation.

### 4.2. Healthcare Industry

In the pharmaceutical and healthcare industries, the prebiotic properties of RBP can significantly improve intestinal diseases and are of great significance to intestinal health. They can also improve cell function in most organs of the human body. Therefore, the development of various new RBP drugs is of great value [88]. In the production of food-grade RBP health supplements, arabinogalactan has been the most extensively studied. The RIG-1, MDA5, ISG-15, and MX1 genes form the frontline of the body’s antiviral immune system [83]. MGN-3, as an immune modulator, can upregulate the expression of these genes, enhance cellular immunity against viral RNA, and achieve anti-aging and antiviral effects. It has been developed as an arabinogalactan supplement to enhance the innate resistance and immunity of the elderly [83]. However, long-term follow-up data are lacking, and further research is needed to validate these findings. In regulating intestinal health, Mihiri [117] summarizes that arabinoxylans derived from plants promote the fermentation of intestinal bacteria and produce short-chain fatty acids that are beneficial to human health. Although one study reported that MGN-3 supplementation increased cytotoxic T cell production and immune responsiveness in healthy subjects, no direct evidence for anti-COVID-19 effects has been demonstrated, and such claims require validation in properly designed clinical trials [118].

In terms of antidepressant effects, animal experiments using rice bran supplement analogs showed good antidepressant symptoms, enhanced mice’s preference for sucrose, and shortened the duration of prolonged sitting in mice [119]. However, their use as direct therapeutic agents in conventional medicine remains investigational, and no RBP has been approved as a drug by major regulatory agencies. In terms of antioxidant properties, RBPs have been applied to the delivery capacity of plasmid DNA, with polyethyleneimine showing better safety and higher infection efficiency [34]. Wang [120] discussed the physical modification of polysaccharides using noncovalent forces like hydrophobic interactions to alter their internal structure, thereby facilitating drug encapsulation and target recognition. In addition, the complex formed by RBP and iron showed higher gene transfer efficiency [95]. Moreover, challenges such as gastric degradation, poor oral bioavailability, and lack of standardized active ingredients continue to hinder their translation into mainstream pharmaceutical products. Therefore, while RBP-based products are commercially available as dietary supplements, their use as registered drugs requires substantially more rigorous evidence.

**Figure 6:**
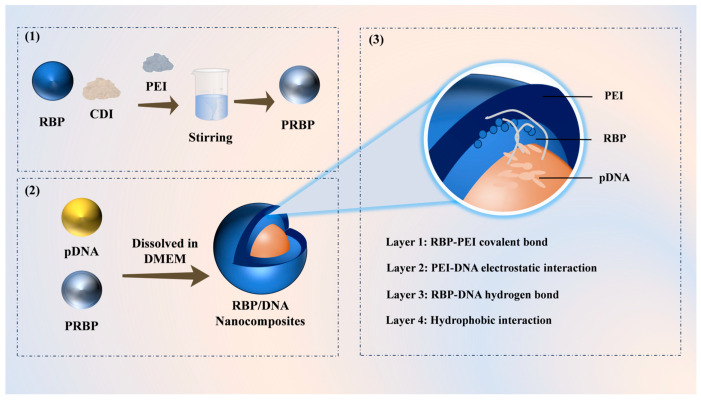
Interactions in the PRBP/DNA complex. Note: (1) The PRBP preparation process. (2) Preparation of RBP/DNA nanoparticles. (3) Internal linking methods for the nanoparticles.

With the development of nanoparticle technology, these issues having been addressed, improving the performance of RBP (Figure 6). Currently, Wu et al. [92] prepared polyethyleneimine-modified rice bran polysaccharide carriers by conjugating low-molecular-weight polyethyleneimine with rice bran polysaccharides. However, during the processing and production of RBP-based health supplements, the limited number of hydrophobic groups and ionic groups within the molecular chains of RBP restricts their industrial application as a functional active substance delivery system in the pharmaceutical field. Currently, further modification and processing are needed in the future to achieve the industrial production of RBP-based pharmaceutical products.

### 4.3. Preservation in Food Industry

In recent years, the film-forming properties, antioxidant properties, and antibacterial properties of plant polysaccharide-based materials have led to their development as food-grade preservation films. These films have excellent coating properties and can be used to preserve foods that are sensitive to oxygen or resistant to bacteria [121]. The coating of RBP is mainly based on microencapsulation, cross-linking, and liposome technology. Bangar [122] showed that adjacent AX chains form a dense macromolecular network due to hydrogen bonds, thereby reducing internal mobility and improving the oxygen barrier properties of the packaging film. The ferulic acid oligosaccharides in rice bran can effectively inhibit Clostridium perfringens, thereby preserving grass carp meat [96]. In addition, Wang confirmed that the addition of RBP enhances the film’s roughness, tensile strength, and antioxidant properties. During industrial production, coating the film helps reduce fruit weight loss and enhances preservation and freshness. During the addition process to the film, RBPs undergo hydrogen bonding interactions within a certain concentration range, reducing the film’s light transmittance, restricting the polymer chain structure, increasing the film’s roughness, tensile strength, and antioxidant activity, but decreasing the film’s water contact angle, thereby protecting the freshness of the fruit [89]. Ó Benito-Román [123] attempted to improve the stability and emulsifying properties of rice bran oil by using a coating material made from maltodextrin and pea protein. The results were not ideal, but better outcomes may be achieved by spray-drying polysaccharides to enhance encapsulation efficiency, which could contribute to research in the field of RBP encapsulation wall materials. Meanwhile, multilayer stacking technology in industrialization can also be used for thin-film packaging of RBP. Li et al. [124] used various polysaccharides to prepare films that not only significantly improved the preservation of blueberries but were also biodegradable, solving the problems of non-biodegradable packaging films causing waste pollution and opening up a new direction for the film industry.

### 4.4. Cosmetics Industry

In the chemical industry, RBPs are predominantly utilized in cosmetic formulations, particularly within active ingredient delivery systems, to augment the antioxidant and anti-aging properties of cosmetic components. Cai et al. [125] explored the application of rice bran non-starch polysaccharides in Pickering emulsions, which are noted for their high safety profile, fine droplet formation, uniform distribution, and capacity to create a dense physical barrier on the skin’s surface. These emulsions can function as natural emulsifying agents in skincare products. Yang et al. [32] investigated the polysaccharides present in rice fermentation products and discovered that, under the synergistic influence of multiple components, these polysaccharides can adsorb moisture, thereby enhancing skin hydration. Furthermore, beta-glucan, a compound found in grains, exhibits soothing and antioxidant properties that contribute to overall skin health and can be employed in skin health maintenance [126]. These findings demonstrate the multifunctional utility of rice bran-derived polysaccharides in cosmetic science, where their roles in emulsion stabilization, hydration enhancement, melanin inhibition, and antioxidant delivery synergistically contribute to developing high-performance, naturally sourced skincare formulations.

### 4.5. Industrial Challenges and Considerations

Previous chapters have discussed the industrial applications of RBP in fields such as food production. However, before this laboratory-scale research can be scaled up for mass production, it must overcome a series of industrial challenges.

In terms of economic feasibility, on the one hand, components in RBP such as cellulose and xylan can serve as inducers, promoting the growth and enzyme secretion of cellulase- and xylanase-producing fungi, thereby enhancing industrial production value [127]. On the other hand, the production cost of RBP depends largely on the extraction and processing methods employed. For example, the enzymatic extraction process for RBP further increases input costs [128]. For baked products, processing complexity and production cost remain important factors that may limit large-scale commercialization. Similarly, in the healthcare sector, RBP-based products are typically marketed as dietary supplements rather than as low-cost raw materials [83]. Furthermore, the development of RBP is currently constrained by the high production costs and complex processing and quality control requirements associated with modified RBP formulations, such as MGN-3 [118].

In terms of scalability, processing methods such as ultrasonication are relatively easy to scale up for RBP extraction and modification. In the food industry, multilayer stacking technologies for RBP-based packaging films have been proposed, indicating the technology’s potential for industrial applications [124]. Enzyme-assisted and fermentation-based modification methods, including those utilizing microorganisms such as Grifola frondosa and Lentinus edodes, have been successfully implemented at the laboratory and pilot scales [20,129]. However, maintaining the stability of enzyme activity and controlling reaction parameters remain technical challenges which limit the implementation of large-scale production. Chemical modification methods, such as the selective oxidation of the hydroxyl group at the C6 position, although scalable, require strict consistency in the production process for each batch [52].

From regulatory and safety perspectives, currently, no RBP-based formulations have been approved as drugs by major regulatory agencies such as the U.S. Food and Drug Administration (FDA) or the European Medicines Agency (EMA). For applications as food additives, RBP-based products must meet the Generally Recognized as Safe (GRAS) standard or obtain Novel Food approval, a process that requires extensive safety data.

In terms of sustainability and other aspects, rice bran is an agricultural byproduct generated during the rice milling process; its use in RBP production aligns with the concept of circular sustainability. Furthermore, RBP-based packaging films possess biodegradable properties, offering an environmentally friendly alternative to synthetic plastics and thereby mitigating pollution-related issues [89,124]. In the cosmetics industry, RBP can serve as a natural emulsifier, thereby reducing reliance on synthetic surfactants [125]. However, current extraction processes often consume large amounts of water and organic solvents. These issues should be addressed in future process development through water recycling, reduced solvent use, and the adoption of energy-saving technologies.

### 4.6. Future Perspectives and Knowledge Gaps

Despite the promising potential of RBP-based products, several gaps remain that hinder their practical application. First, the relationship between activity and structure is not yet clear; future research should employ activity-guided isolation methods to identify effective bioactive components. Second, the in vivo metabolic processes of these products have hardly been studied. Third, there are currently no standard reference materials or quality standards applicable to these products, which impedes regulatory approval. Fourth, the technical and economic feasibility of green processing methods and scalable production processes for these products requires further evaluation. Fifth, the integration of these products with emerging applications, such as smart packaging, has not yet been fully explored. Therefore, larger-scale clinical trials and related experimental studies are needed in the future.

## 5. Conclusions

The effective utilization of RBP depends on a deeper understanding of the relationships among its structural characteristics, functional properties, and application performance. The functional attributes of RBP are intrinsically linked to its monosaccharide composition and three-dimensional conformation. Variations in molecular weight and the nature of functional groups can significantly influence the applicability of RBP in industries such as food additives. Despite extensive research into the biological functions of RBP, including its anti-inflammatory, antioxidant, and hypoglycemic effects, it has yet to be classified as a dual-purpose substance for both medicinal and nutritional use. Moreover, research on the chemical modifications of RBP has predominantly concentrated on extraction methodologies and physical properties, with limited investigation into the implications of its biological activity and functional characteristics for practical applications. In terms of industrial potential, beyond its prevalent use as animal feed, RBP is increasingly being utilized as a food additive and nutritional supplement in products such as baked goods and beverages to augment their nutritional value. However, the industrial application of RBP currently faces numerous challenges, including high production costs, immature large-scale manufacturing processes, a lack of unified quality standards and regulatory certification, as well as potential safety and environmental sustainability issues associated with chemical modification. Future research should focus on developing green and efficient extraction technologies and establishing standardized quality control systems to advance the practical industrial application of RBP.

## Figures and Tables

**Table 1:** Effects of different extraction methods on RBP.

Variety	Extraction Method	Monosaccharide Composition	Yield	Molecular Weight	Key Biological Activity	Reference
RBP	Hot water extraction	Man-Glc-Gal-Ara	8.73%	0.181–1.562 × 10^4^ Da	DPPH radical were 23.75%	[28]
RBP	Ultrasonic–microwave cooperative extraction	Man-GlcN-Rha-Glc-Xyl-Ara	9.33%	0.153–2.030 × 10^4^ Da	DPPH radical were 42.65%	[28]
RBP	Alkali extraction	Man-Glc-Gal-Xyl-Ara	10.4%	0.137–10.258 × 10^4^ Da	DPPH radical were 37.50%	[28]
RBPI RBPII RBPIII	Ultrasound–enzyme–ultrasound assisted extraction	Glu-Gal-Rha-Man-GalA-Ara-Xyl-GlcA-Rib-Fuc	5.57 ± 0.34%	4.07 ± 0.1 × 10^3^–266.73 ± 1.62 × 10^3^ Da	RBPI: DPPH radical were 27.21% RBPII were 62.07% RBPIII were 55.12%	[36]
WS-DF AS-DF	Cellulase treatment	WS: Glu-Xyl-Ara AS: Glu-Gal-Xyl	WS—5.54% AS—7.22%	——	ABTS radical were 20.78–22.37% Glucose adsorption capacity is 3.43–3.82 mmol/g	[37]
WS-DF AS-DF	Ultrasound treatment	WS: Glu-Xyl-Ara AS: Glu-Gal-Xyl	WS—8.79% AS—9.58%	——	ABTS radical were 14.38–21.69% Glucose adsorption capacity is 3.69–4.61 mmol/g	[37]
WS-DF AS-DF	Combined treatment using cellulase and ultrasound	WS: Glu-Xyl-Ara AS: Glu-Gal-Xyl	WS—9.03% AS—8.14%	——	ABTS radical were 5.75–23.29% Glucose adsorption capacity is 2.68–2.76 mmol/g	[37]
GS-RBP	Fermentation	GS-FRB: D-glu, D-man, D-xyl, L-ara and D-fru GS-DRB: D-glu and D-man	9.67% 9.44% 8.33% 6.33%	4944.84 × 10^3^ Da 2560.64 × 10^3^ Da	The tumor volume in the control group was 5%, while that in the GS-DRB-11 group was 4.26 ± 4.4%	[29]

—— indicates that no report has been made yet.

**Table 2:** Effects of different chemical modifications on RBP.

Variety	Modification Method	Mechanism	Degree of Substitution (DS)	Molecular Weight (kDa)	Key Biological Activity	Reference
Glucan in RBP	Sulfation	Hydroxyl chemical conversion to sulfate ester group.	P444A = 1.6 P445 = 1.8 P445A = 1.9 P445B = 1.7 P445C = 0.6 P446 = 1.7 P446A = 1.3 P446B = 1.2	P444A = 68 P445A = 69.2 P445B = 30.5 P445C = 5 P446A = 58.6 P446B = 27.3	P444, P445, and P446 inhibit the invasion of human cytomegalovirus (HCMV).	[47]
RBP	Sulfation	The hydroxyl groups in polysaccharide molecules are replaced by sulfate ester groups.	0.507 ± 0.02	9.26	From rough flakes to smooth curls. SRBP has stronger antibacterial activity than RBP and inhibits *Escherichia coli*.	[48]
RBP	Carboxymethylation	Carboxymethyl functional groups interact with RBP. These functional groups are introduced into the polysaccharide molecule through physical treatment. Absorption peaks for the COO group appear at 1603 cm^−1^ and 1329 cm^−1^.	0.91	——	As an electron-withdrawing group within the polysaccharide, the carboxyl group contributes to RBP’s antioxidant activity.	[49]
RBP	Acetylation	Functional groups were introduced into the polysaccharide molecules via physical treatment. Absorption peaks for C=O at 1727 cm^−1^ and C-O stretching vibrations at 1253 cm^−1^ were observed.	0.32	——	Compared to unmodified RBP, the ability to scavenge superoxide anions significantly increased to 49.9%.	[49]
RBP	Phosphorylation	Phosphorylated functional groups interact with RBP. Functional groups are introduced into polysaccharide molecules via physical treatment. An absorption peak for P=O appears at 1264 cm^−1^.	0.28	——	The DPPH radical scavenging rate of phosphorylated RBP reached 51.3%, substantially enhancing the DPPH radical scavenging capacity of RBP.	[49]
RBP	Metal complexation	Reaction of RBP with ferric chloride under alkaline conditions.	——	——	The complex exhibits a binding capacity of 7.27 × 10^6^ L/mol and significant gelling properties.	[50]

—— indicates that no report has been made yet.

**Table 3:** Different polysaccharide-based drug delivery systems.

Polysaccharide Variety	Delivery Method	Mechanism	Combined Substance	Characteristics Efficacy	Reference
Arabinoxylan in rice bran	Covalent Hydrogel	Covalent cross-linking	Curcumin	Form quickly, and they are strong and thermostable	[88]
Polyethylenimine-RBP-Fe(III)	Nanoparticles	The DNA-aggregating ability of the complex can be enhanced by metal ions	Polyethylenimine	The PIP complex significantly enhances DNA condensation capacity	[34]
RBP	Film	Solution casting method	Eco-friendly gelatin	Increased the film’s roughness, tensile strength, and antioxidant activity	[89]
RBP	Emulsion	Formation of covalent bonds between polysaccharides and small-molecule peptides	Fish oil	Possesses excellent emulsifying capability and good thermal stability and storage stability	[7]
Rice starch	Film	Hydrogen bond	Tara gum and pectin	Hygroscopic	[90]
*Triticosecale* Wittmack	Electrospinning	High-voltage electrostatic field force	gelatin	With a higher quality mesh structure	[91]

**Table 4:** Summary of functional studies on RBP.

Polysaccharide Component	Characteristics	Mechanism	Reference
RBP	Emulsifying properties	Ultrasonic treatment–enzymatic hydrolysis–re-ultrasonic treatment can expose more hydrophilic sites, providing an ideal environment for the formation of a stable oil–water interface.	[36]
RBP	Stability	Microwave treatment of RBP causes changes in the structure of the modified solution, thereby enhancing its heat resistance and exhibiting thermal stability.	[43]
Rice bran fiber	Thermal stability	After adding rice bran fiber, the gelatinization onset temperature (To) increases.	[95]
Rice bran ferulic acid oligosaccharide	Water-holding capacity	Improving water retention properties, inhibiting the growth and reproduction of microorganisms and extending the shelf life of food.	[96]
Modified RBP	Rheological properties	Combined use can significantly reduce phase separation rates in high-fat, low-pH environments.	[61]
Rice polysaccharide	Rheological properties	Adding 0.5–1.0% RBP can increase the viscosity of rice starch and improve its stability at high temperatures.	[97]
PEI-modified RBP	Gene transfection performance properties	Not only does it have more significant DNA concentration capabilities, but it also has higher gene transfection efficiency.	[98]

## Data Availability

No new data were created or analyzed in this study.

## Abbreviations

The following abbreviations are used in this manuscript:

RBP	Rice bran polysaccharide
FAO	Food and Agriculture Organization
DS	Degrees of substitution
iNOS	Inducible Nitric Oxide Synthase
DSS	Dextran Sulfate Sodium
NK	Natural killer
ICAM-1	Intercellular adhesion molecule-1
MGN-3	Biobran
RBAC	Rice bran arabinoxylan compound
PEI	Polyethyleneimine
OSA	Octenylsuccinic anhydride
COVID-19	Coronavirus disease 2019
FDA	Food and Drug Administration
EMA	European Medicines Agency
GRAS	Generally Recognized as Safe
